# Sustainability of evidence-based practices in the management of infants with bronchiolitis in hospital settings - a PREDICT study protocol

**DOI:** 10.1186/s12913-022-08450-z

**Published:** 2022-08-29

**Authors:** Victoria Ramsden, Franz E. Babl, Stuart R. Dalziel, Sandy Middleton, Ed Oakley, Libby Haskell, Anna Lithgow, Francesca Orsini, Rachel Schembri, Alexandra Wallace, Catherine L. Wilson, Elizabeth McInnes, Peter H. Wilson, Emma Tavender

**Affiliations:** 1 Australian Catholic University, 40 Edward Street, North Sydney, NSW 2060 Australia; 2 Emergency Research, Murdoch Children’s Research Institute, The Royal Children’s Hospital, Level 4 West, 50 Flemington Road, Parkville, VIC 3052 Australia; 3 University of Notre Dame, 160 Oxford Street, Darlinghurst, NSW 2010 Australia; 4 Emergency Department, The Royal Children’s Hospital, 50 Flemington Road, Parkville, VIC 3052 Australia; 5 Departments of Paediatrics and Critical Care, The University of Melbourne, Grattan Street, Parkville, VIC 3010 Australia; 6 Departments of Surgery and Paediatrics, Child and Youth Health, University of Auckland, 28 Park Road, Grafton, Auckland, 1023 New Zealand; 7 Children’s Emergency Department, Starship Children’s Hospital, 2 Park Road, Grafton, Auckland, 1023 New Zealand; 8 Nursing Research Institute, St Vincent’s Health Network Sydney, St Vincent’s Hospital Melbourne, and Australian Catholic University, Sydney, Australia; 9 St Vincent’s Hospital, Level 5 DeLacy Building, Victoria Road, Darlinghurst, NSW 2010 Australia; 10 Royal Darwin Hospital, 105 Rocklands Dr, Tiwi, Northern Territory 0810 Australia; 11 Clinical Epidemiology and Biostatistics, Melbourne Children’s Trials Centre, Murdoch Children’s Research Institute, The Royal Children’s Hospital, 50 Flemington Road, VIC 3052 Parkville, Australia; 12 Department of Paediatrics, Waikato Hospital, Selwyn St, Hamilton Central, Hamilton, 3204 New Zealand; 13 Australian Catholic University, Building 460, Level 8, 250 Victoria Parade, East Melbourne, Victoria 3002 Australia

**Keywords:** Sustainability, Sustainment, implementation science, evidence-based practice, Bronchiolitis, Acute care, Paediatric, Emergency medicine

## Abstract

**Background:**

Understanding how and why de-implementation of low-value practices is sustained remains unclear. The Paediatric Research in Emergency Departments International CollaboraTive (PREDICT) Bronchiolitis Knowledge Translation (KT) Study was a cluster randomised controlled trial conducted in 26 Australian and New Zealand hospitals (May-November 2017). Results showed targeted, theory-informed interventions (clinical leads, stakeholder meetings, train-the-trainer workshop, targeted educational package, audit/feedback) were effective at reducing use of five low-value practices for bronchiolitis (salbutamol, glucocorticoids, antibiotics, adrenaline and chest x-ray) by 14.1% in acute care settings. The primary aim of this study is to determine the sustainability (continued receipt of benefits) of these outcomes at intervention hospitals two-years after the removal of study supports. Secondary aims are to determine sustainability at one-year after removal of study support at intervention hospitals; improvements one-and-two years at control hospitals; and explore factors that influence sustainability at intervention hospitals and contribute to improvements at control hospitals.

**Methods:**

A mixed-methods study design. The quantitative component is a retrospective medical record audit of bronchiolitis management within 24 hours of emergency department (ED) presentations at 26 Australian (*n* = 20) and New Zealand (*n* = 6) hospitals, which participated in the PREDICT Bronchiolitis KT Study. Data for a total of 1800 infants from intervention and control sites (up to 150 per site) will be collected to determine if improvements (i.e., no use of all five low-value practices) were sustained two- years (2019) post-trial (primary outcome; composite score); and a further 1800 infants from intervention and control sites will be collected to determine sustained improvements one- year (2018) post-trial (secondary outcome). An a priori definition of sustainability will be used. The qualitative component will consist of semi-structured interviews with three to five key emergency department and paediatric inpatient medical and nursing staff per site (total *n* = 78-130). Factors that may have contributed to sustaining outcomes and/or interventions will be explored and mapped to an established sustainability framework.

**Discussion:**

This study will improve our understanding of the sustainability of evidence-based bronchiolitis management in infants. Results will also advance implementation science research by informing future de-implementation strategies to reduce low-value practices and sustain practice change in paediatric acute care.

**Trial registration:**

Australian and New Zealand Clinical Trials Registry No: ACTRN12621001287820.

Contributions to the literature

- This will be one of the few studies examining sustainability (continued receipt of benefits) and sustainment (continued use) of an intervention that improved evidence-based practices in acute care settings.
- Findings will contribute knowledge of the factors that improve the sustainability of evidence-based management of infants with bronchiolitis.
- Understanding effective sustained implementation strategies will inform future efforts to improve practice in acute care settings as well as future implementation research.

## Background

In infants less than 1 year of age, the most common lower respiratory tract infection is bronchiolitis [1]. Bronchiolitis is caused by a viral infection, most frequently by respiratory syncytial virus and can be life-threating [2, 3]. In Australia and New Zealand, bronchiolitis is the leading cause of hospital admissions in infants under 6 months of age [2, 4, 5] accounting for 56% of Australian hospital admissions in infants less than 12 months of age, 7-9% of New Zealand hospital admissions in children 0 to 14 years of age [5–7], with infants from indigenous and deprived populations being most at risk [8].

**Fig. 1:**
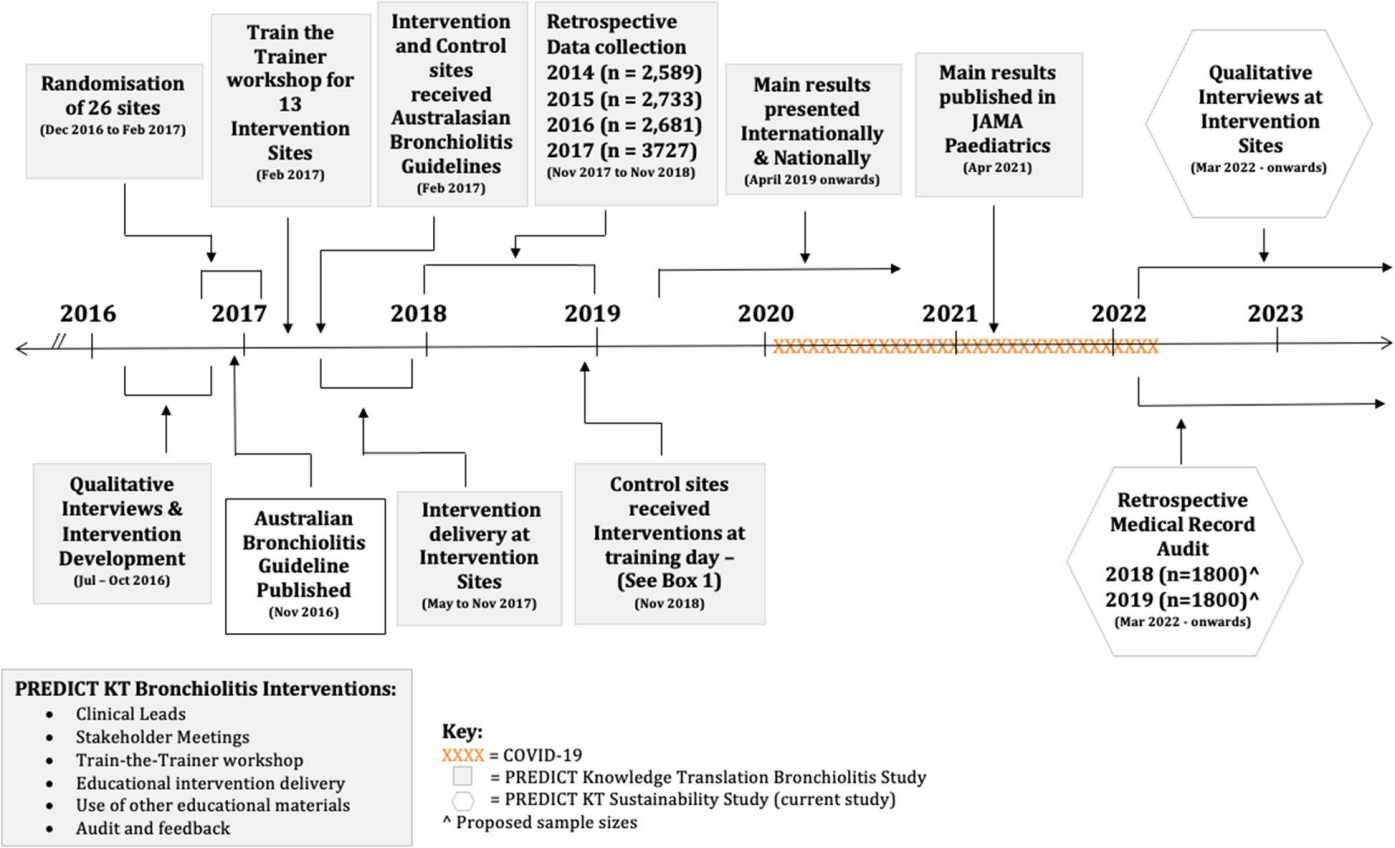
Timeline from PREDICT KT Study to the PREDICT KT Sustainability Study

Recommended management of bronchiolitis is well defined, comprising of supportive practices such as respiratory and hydration support [2, 3, 9]. There is strong evidence that five therapies and management processes, namely salbutamol, glucocorticoids, antibiotics, adrenaline and chest x-rays (CXRs) are ineffective or ‘low value’ and should not be used in the management of infants with bronchiolitis [1, 5, 10, 11]. Current international clinical practice guidelines, including the 2016 evidence-based guideline from Australia and New Zealand (Australasian Bronchiolitis Guideline) [1, 5] recommend against their use. Yet, variations in practice persist. An Australasian retrospective audit conducted at seven hospitals found that in 3856 infants who presented with bronchiolitis, 27-48% received at least one of the five low-value practices [12]. These results are consistent with the literature [4, 13], showing that even when evidence reveals no benefits, abandoning (i.e. de-implementing) low-value practices is often harder than implementing new practices [14–16]. In response to the variations in practice, we undertook the Paediatric Research in Emergency Departments International CollaboraTive (PREDICT) Bronchiolitis Knowledge Translation (KT) Study to improve bronchiolitis care. It demonstrated that use of targeted, theory-informed interventions significantly improved bronchiolitis care by de-implementing these five low-value practices by 14.1% (adjusted risk difference, 95% CI, 6.5-21.7%; *P* < .001) [4]. The focus of this current study is to determine if the change has been sustained (Fig. 1).

### Sustainability: definition and overview of research

Sustainability studies are rare, with the few undertaken reporting poor long-term (sustained) compliance [17, 18]. This area of research is complicated by no clear or consistent definition of sustainability [19, 20]. Common elements of sustainability have been identified, namely, continued capacity to deliver the intervention, continued provision of the implementation intervention, and ongoing clinical benefit from the intervention [21]. Additionally, there is debate about distinguishing between *sustainability* and *sustainment* [22]. For our purposes, we define sustainability as the degree to which an evidence-based innovation, after external support has ceased, can deliver its benefits as intended over a prolonged period of time i.e. sustained benefits [17, 23]. We define sustainment as the continued use of an implementation intervention i.e. its sustained use [17, 23].

To date, measurement of the ongoing effect of implementation research largely reports success by the ‘clinical outcomes’ that the intervention was designed to change (i.e. sustainability of improved clinical care) [20]. Often there is insufficient reporting on and evaluation of continuation of the process or implementation of the evidence-based intervention (i.e. sustainment) that was used to bring about the change (i.e. sustainment of education delivery or use of clinical champions etc) [18, 20]. Yet systematic reviews assessing the sustainability of effective behaviour change studies with multiple behaviour change components found 60% maintained implementation of only one component of the proven intervention [19, 24, 25]. Thus the measurement of both sustainability and sustainment are important for the long-term understanding of implementation interventions [26]. For the purpose of this study, we define sustainability as the sustained benefits, i.e. the continued clinician behaviour change (no use of the five low-value bronchiolitis practices) [27]. We define sustainment as the continued use of the targeted theory-informed bronchiolitis implementation strategy [23, 27].

### Sustainability frameworks

A wide variety of terms, (i.e. routinisation, durability or institutionalisation) have previously been used when discussing sustainability [28]. However, these terms are rarely operationalised or defined conceptually [28]. Therefore, understanding factors that contribute to sustained interventions is vital. The use of sustainability frameworks can help determine the causal factors which lead to the success or failure of an intervention allowing for future studies to adopt better strategies [29, 30]. Yet the use of sustainability frameworks in research is lacking [31]. Whilst various frameworks have been identified as relevant for sustainability, few have been tested to determine if they are good predictors of sustainability [28, 31]. Furthermore, few frameworks are designed specifically for acute care settings and there is no consensus for which framework is most relevant [30, 31]. Therefore there is a need to test these frameworks to determine applicability in specific settings [28].

#### The PREDICT Bronchiolitis KT Study

The ‘Knowledge Translation (KT) in Australasian Paediatric Acute Care Settings Study’ (PREDICT Bronchiolitis KT study) (ACTRN12616001567415) was a multi-centre, cluster randomised controlled trial conducted in 26 Australian and New Zealand hospital Emergency Departments (EDs) and paediatric inpatient areas during the 2017 Southern Hemisphere bronchiolitis season (1st May to 30th November 2017), involving 3240 infants [4]. Of the 26 hospitals, 13 were randomised to the intervention group and 13 to the control group with all hospitals completing the trial. The intervention group received targeted, theory-informed interventions developed to address factors identified as influencing bronchiolitis management in Australia and New Zealand [32]. Interventions consisted of: identification of stakeholder meetings; clinical leads; Train-the-trainer workshop; educational materials with scripted, targeted messaging; other educational material consisting of a clinician video demonstrating how to talk with families about bronchiolitis, promotional materials, evidence fact sheets; and finally monthly audit and feedback (Table 1) [4, 33]. Both intervention and control group hospitals received printed and electronic copies of the Australasian Bronchiolitis Guideline.

**Table 1:** Implementation Strategy Components used in the PREDICT Bronchiolitis KT Study [4, 33]

Intervention Components	Intervention Hospitals	Control Hospitals Post Trial^a^
Clinical leads	Four clinical leads for the duration of the study, included a medical and a nursing lead from each emergency department and inpatient paediatric areas. Key tasks included attending a 1-day train-the-trainer workshop, spearheading educational intervention and other educational materials delivery to all staff, supervising monthly audit completion and delivery of feedback, and management of study requirements.	Encouraged to allocate a medical and nursing lead, but no further guidance provided.
Stakeholder Meetings	Study team presented the Australasian Bronchiolitis Guideline to clinical leads, discussed local and international bronchiolitis management variations, reviewed results of the local audit, and discussed any local anticipated barriers, with the aim to gain site buy-in.	Nil.
Train-the- trainer workshop	1-day workshop for clinical leads: discussed Australasian Bronchiolitis Guideline and evidence supporting recommendations, qualitative study identifying the facilitators and barriers of bronchiolitis management, implementation, and the development of interventions. Clinical leads received demonstrations on how to deliver educational interventions to their staff, outlines of study data timelines and requirements, and enabled planning time for clinical leads.	One-day workshop providing bronchiolitis intervention materials, with up to four clinical leads (medical and nursing) invited to attend. Discussion on what the aims of education materials were. Individual hospital data from the cluster RCT was presented (2014-2017). Feedback in relation to similar hospitals.
Educational intervention delivery	Key findings from qualitative study were presented in a PowerPoint with scripted messages stating use of behaviour change techniques most likely to effect change. Clinical Leads oversaw education delivery to medical and nursing staff using PowerPoint presentation. Within first month, aimed to educate 80% of staff and to ensure all staff educated ongoing education provided throughout duration of study.	PowerPoint presentation which was the same as presentation provided to the intervention hospitals.
Use of other educational materials	Clinical leads locally delivered promotional materials, evidence factsheets, clinician training video, and parent/caregiver information.	Education materials that were provided to the intervention hospitals.
Audit and feedback	Monthly audits of the first 20 bronchiolitis presentations, with report providing comparison between individual hospital results and top-performing site. Clinical leads disseminated report to their staff in written and verbal format; action planning with target setting encouraged.	Audit form and own hospital results from trial (4 years of data).
Ongoing facilitation	Weekly contact between clinical leads and study leads. Opportunities to ask questions via email.	Nil.

^a^delivered at trial completion in November 2018

Findings of the PREDICT Bronchiolitis KT Study demonstrated that, when compared with control hospitals, intervention hospitals significantly improved adherence in management of bronchiolitis during the acute care period (first 24 hours of hospital presentation) for the five key low-value practices as per Australasian Bronchiolitis Guideline recommendations (14.1% adjusted risk difference, 95% CI, 6.5-21.7%; *P* < .001) [4, 34]. Specifically, during the acute care period (first 24 hours of hospital presentation), adherence to the Australasian Bronchiolitis Guideline recommendations with no use of salbutamol, glucocorticoids, antibiotics, adrenaline and CXRs, was 85% (95% CI, 82.6-89.7%) in the intervention group hospitals compared with 73% (95% CI, 65.3-78.8%) in the control group hospitals [4, 34]. At trial completion (November 2018), up to four nurses and doctors from each of the 13 control hospitals attended a 1 day workshop where they were given training and provided with intervention materials (Table 1). However, no on-going facilitation was provided.

#### Aim

The aim of this mixed methods study is to: i) determine if the use of the targeted theory-informed interventions from the PREDICT Bronchiolitis KT Study have been effective at sustaining improvements (adherence to the Australasian Bronchiolitis Guideline i.e. no use of salbutamol, glucocorticoids, antibiotics, adrenaline and CXRs) in the care of children with bronchiolitis during the acute care period (first 24 hours of hospital presentation including ED and paediatric inpatient unit care) 1 and 2 years post-trial completion, specifically in 2018 and 2019, at intervention group hospitals; ii) determine if there are any improvements to adherence to the Australasian Bronchiolitis Guideline in control group hospitals; iii) understand the factors that influence the sustainability of improvements in intervention group hospitals and; iv) explore factors which may have contributed to improvements at control group hospitals. We will use an a priori definition of sustainability (see Data analysis below).

## Methods

### Study design

A mixed-methods study design will be used. Retrospective medical record audits will assess the sustainability of the improvements, and qualitative semi-structured individual or group interviews will assess the sustainment of the interventions.

### Setting/hospital selection

The original twenty-six hospitals (Australia = 20 and New Zealand = 6) which participated in the 2017 PREDICT Bronchiolitis KT Study will be invited to participate [4]. Hospitals must be able to meet local governance requirements and collect the required data regarding bronchiolitis care from the medical records within a specified timeframe (e.g. 6 months following local governance approval).

Clinical Directors of EDs or general paediatric inpatient units whose hospital participated in the PREDICT Bronchiolitis KT Study will be approached by email to assess interest and capacity for their hospitals to be involved in this study [4]. The research team will send an invitation email, an information statement, and an expression of interest form to eligible hospitals. Clinical Directors will be encouraged to discuss the study with relevant personnel in their departments. Phone and email contact will be made by the research team approximately 1 week after sending the recruitment pack to check receipt and ascertain interest. A site meeting will be arranged (via face-to-face or telephone) with relevant stakeholders in the ED and paediatric inpatient unit to discuss details and logistics of the study. Hospitals will only be able to participate in the qualitative semi-structured individual or group interviews if they have agreed to conduct the retrospective medical record audit.

#### Quantitative study

##### Selection of patients with bronchiolitis

A retrospective patient medical record audit will be conducted by designated site study staff at each consenting site, under the supervision of site Principal Investigators. A list of all infants who presented between 1 January 2018 and 31 December 2019 with International Classification of Diseases 9th and 10th revision (ICD-9 or ICD-10) discharge codes for bronchiolitis (Table 2) will be retrospectively identified. To be eligible for inclusion, patients must be: aged less than 1 year (at time of presentation); AND have both an ED and discharge diagnosis of bronchiolitis. There are no patient-level exclusion criteria.

**Table 2:** International Classification of Diseases (ICD) codes used for identifying patients

ICD code		Description
ICD–10	J21	Acute bronchiolitis
	J21-0	Acute bronchiolitis due to respiratory syncytial virus
	J21-1	Acute bronchiolitis due to human metapneumovirus
	J21-8	Acute bronchiolitis due to other specified organisms
	J21-9	Acute bronchiolitis, unspecified
ICD-9	466-11	Acute bronchiolitis due to respiratory syncytial virus
	466-19	Acute bronchiolitis due to other infectious organisms

Data for a total of 1800 infants (approximately 150 infants per site) will be collected at 2 years post-trial (2019), to address the primary outcome. Similarly, data from 1800 infants will be collected at one-year post-trial (2018). If a site has less than 150 infant presentations per year, 100% of eligible infants will be selected. If a site has more than 150 infant presentations per year, a random sample of 150 infants per year will be selected by an independent statistician using computer software.

#### Outcome measures for quantitative study

Primary Outcome:

1. Adherence with all five Australasian Bronchiolitis Guideline Recommendations (composite outcome) known to have no benefit (salbutamol, glucocorticoids, antibiotics, adrenaline and CXR) when an infant presents with bronchiolitis in the acute care period (first 24 hours of hospitalisation) at two- years (2019) following the completion of the PREDICT Bronchiolitis KT Study in intervention hospitals [34];

Secondary Outcomes:

2. Adherence with all five Australasian Bronchiolitis Guideline Recommendations (composite outcome) known to have no benefit (salbutamol, glucocorticoids, antibiotics, adrenaline and CXR) when an infant presents with bronchiolitis in the acute care period (first 24 hours of hospitalisation) at one- year (2018) following the completion of the PREDICT Bronchiolitis KT Study in intervention hospitals [34];
3. Adherence with all five Australasian Bronchiolitis Guideline Recommendations (composite outcome) known to have no benefit (salbutamol, glucocorticoids, antibiotics, adrenaline and CXR) when an infant presents with bronchiolitis in the acute care period (first 24 hours of hospitalisation) at 3a) one- year (2019) following provision of study materials, study results, and ‘Train the Trainer’ education (composite of all five practices) [4, 34]; and 3b) one- year (2018) following the completion of the PREDICT Bronchiolitis KT Study in control hospitals [4, 34].

The periods of one- and two- years following delivery of the intervention will be measured from the post-trial completion date of 30th November 2017.

### Data collection procedure

#### Medical record audit

Routinely collected bronchiolitis management data will be retrospectively extracted from medical records by designated site study staff. De-identified data extracted from the medical records will be entered into a standardised form in a Research Electronic Data Capture (REDCap) database by designated study staff at each hospital.

#### Sample size

The sample size for this medical record audit will depend on the number of participating hospitals and will be recalculated upon completion of the hospital recruitment stage. Based on our previous study [4], assuming an Intra-cluster Correlation Coefficient of 0.11, an expected proportion of no use of all five low-value practices) of 85% (based on results of the PREDICT Bronchiolitis KT Study) [4] and a Coefficient of Variation of cluster sizes of 0.16 [4], a total sample size of 1800 (obtained from 12 intervention hospitals (clusters) with an average size of 150 infants) will achieve a confidence interval (CI) half-width of 7% (95%CI: 78 to 92%). An equivalent number of infants, i.e. 1800 in total, will also be obtained from ~ 12 control hospitals (clusters) with an average size of 150 infants.

### Data analysis

Analysis of the data from the medical record audit will occur at the patient level, according to the group (intervention/control) that hospitals were randomly allocated in the previous study [4]. Absolute and relative frequencies of no use of all five low-value practices will be calculated. Means and standard deviations or medians and interquartile range will be used to present continuous outcomes. The proportions of no use of all five low-value practices and its 95% confidence intervals will be calculated and presented by study group. *Exploratory Objectives* will be run to estimate the marginal differences in proportions of no use of all five low-value practices between the study groups, using Generalised Linear Mixed (GLM) [34]. If the GLM models do not converge, logistic regression with a clustered-robust standard error (hospital level) will be used to calculate odds ratios. *Exploratory Objectives* will examine adherence to the five individual practices (i.e, no use of all five low-value practices); difference between intervention and control groups in 2019; difference between intervention and control groups in 2018; and trends over time (Table 3).

**Table 3:** Exploratory objectives

To determine:	
• The proportion of infants presenting to hospital with bronchiolitis in the acute care period^a^, at intervention group hospitals, who received care that adhered with each of the five individual Australasian Bronchiolitis Guideline Recommendations known to have no benefit^b^: a) two- years (2019) following delivery of a targeted intervention [34]; and b) one- year (2018) following delivery of an intervention designed to promote evidence-based practice adherence [34];	
• The proportion of infants presenting to hospital with bronchiolitis in the acute care period^a^, at control group hospitals, who received care that adhered with each of the five individual Australasian Bronchiolitis Guideline Recommendations known to have no benefit^b^: a) two- years (2019) following delivery of an intervention designed to promote evidence-based practice adherence [4, 34]; and b) one- year (2018) following delivery of bronchiolitis intervention [4, 34];	
• The difference between control and intervention group hospitals in the proportion of infants presenting to hospitals with bronchiolitis who received care that adhered with each of the five individual Australasian Bronchiolitis Guideline Recommendations known to have no benefit^b^ in the acute care period: a) two- years (2019); following the completion of the PREDICT Bronchiolitis KT Study [4, 34]; and b) one- year (2018); following the completion of the PREDICT Bronchiolitis KT Study [4, 34];	
• The difference between control and intervention group hospitals in the proportion of infants presenting to hospital with bronchiolitis who received care that adhered with all five Australasian Bronchiolitis Guideline Recommendations known to have no benefit^b^ in the acute care period: a) two- years (2019) following the completion of the PREDICT Bronchiolitis KT Study (composite outcomes of all five practices) [4, 34]; and b) one- year (2018) following the completion of the PREDICT Bronchiolitis KT Study (composite of all five practices) [4, 34];	
• The trends in the proportions of infants presenting to hospital with bronchiolitis who received care that adhered with each of the individual five Australasian Bronchiolitis Guideline Recommendations known to have no benefit^b^ yearly from 2014 to 2019 in the acute care period, at control and intervention group hospitals [34].	

^a^ First 24 hours of hospitalisation, ^b^ no use of: i) salbutamol, ii) glucocorticoids, iii) antibiotics, iv) adrenaline, and v) chest x-ray

An a priori definition of sustained improvements will be based on the 7% median improvement achieved in the PREDICT Bronchiolitis KT Study [4]. Practices will be defined as sustained when there is either a maximum of a seven percentage point decrease or any level of improvement (i.e. -7% to infinity) from the participating hospital’s individual post-implementation results based on a composite outcome of the five low-value practices of interest [4, 34].

#### Qualitative study

##### Recruitment of clinical staff for interviews

The Clinical Director of either the EDs or paediatric inpatient areas of the participating hospital will be approached by email to confirm their hospital’s participation in the qualitative interviews. The Clinical Director (or site Principal Investigator) will be requested to send participant information forms to eligible medical and nursing staff. Phone or email contact with the Clinical Directors or site Principal Investigator will be made by the research team approximately 1 week after sending the recruitment pack to check receipt and facilitate recruitment.

Qualitative group interviews will be undertaken with a purposive sample of nurses and doctors at each hospital who treat infants with bronchiolitis in participating EDs and paediatric inpatient areas. These will be undertaken at intervention and control group hospitals at each site or via telephone/video interviews. Where it is not possible to undertake semi-structured group interviews at the hospital due to logistical issues or COVID-19 restrictions, interviews will be undertaken with individual staff members. To be eligible for interview, participants must be: on an active practice roster; working full-time or part-time in a clinical role as either registered nurses or doctors who care for children with bronchiolitis in New Zealand or Australian EDs or paediatric inpatient areas. The original PREDICT Bronchiolitis KT Study clinical leads will be included in the sample, where possible [4]. Bank or agency staff (nurses); interns/students, locums (medical); and clinicians not currently engaged in clinical practice will be excluded.

#### Outcome measures for qualitative study

- Factors that contributed to sustainability/non-sustainability of improvements (i.e., no use of the five low-value practices) at intervention group hospitals 4 years post-trial completion including the sustainment of PREDICT Bronchiolitis KT Study implementation strategy;
- Factors that contributed to improvements (i.e. no use of the five low-value practices) or deterioration at control group hospitals 4 years post-trial completion, including the use of PREDICT Bronchiolitis KT Study implementation strategy;
- Fidelity to and adaptation of the PREDICT Bronchiolitis KT Study implementation strategy at intervention and control group hospitals 4 years following intervention delivery (2018, 2019, 2020, 2021) [4, 34].

### Data collection procedure

#### Clinician interviews

Eligible ED and paediatric inpatient area staff will contact the research staff directly, by email or phone, so a convenient date, time and location for the individual or group semi-structured interview can be arranged. Participants will be interviewed face-to-face or by video link (e.g., ZOOM), depending on the participants’ preferences and convenience. Consent will be implied if an eligible interested individual presents at the agreed upon time to participate in the semi-structured interview. The interviews will be conducted for approximately 30-45 minutes and will be digitally recorded using a voice recorder or via zoom (all recordings will be saved electronically to the Murdoch Children’s Research Institute secure platform). Verbal confirmation will be obtained for consent to audio-tape the interview.

#### Interview schedule and conduct

The interview schedule has been developed by the research team, which includes emergency physicians, nurses and researchers. The schedule includes open-ended questions to elicit participant’s views on how the PREDICT Bronchiolitis KT Study was perceived by staff; use and adaption of intervention materials; and potential barriers and enablers to sustaining practice change. The interview schedule will be piloted to ensure face and content validity.

#### Sample size

A maximum of three to five individuals per site (*n* = 78 to 130) from a cross section of nursing and medical staff from both ED and paediatric inpatient areas will be invited to participate in the interviews.

### Data analysis

Thematic analysis will be performed on qualitative transcribed interview data imported into NVIVO10 (QSR International Pty Ltd., Australia), using an iterative process and open coding relating extracts of text to key questions using saliency analysis i.e., the extent to which each code occurs, and implied importance (regularly mentioned or considered important by researchers or participants) [35]. Hospitals will be compared and contrasted by intervention and control group allocation from the PREDICT Bronchiolitis KT Study. Text will be cross indexed when it is relevant to more than one theme. Codes will be collapsed and decreased as analysis progresses. Cross-checking of the coding framework and discrepancies will be conducted by a second researcher and discussed to reach consensus. Participant quotes will be linked to the final agreed set of themes and sub-themes. When no new insights or issues between participant groups or between hospitals are reported over three consecutive interviews, data will be deemed saturated [36]. Themes will then be mapped to a sustainability framework(s) [23, 29, 37].

## Discussion

This study aims to evaluate if targeted, theory-informed interventions sustain improvements in bronchiolitis care (i.e. no use of the five low-value practices: salbutamol, glucocorticoids, antibiotics, adrenaline and CXRs) in the acute care period (first 24 hours of hospital presentation) in EDs and paediatric inpatient units, two-years (2019) after their implementation [34]. Results will help to inform the content and format/methods by which to deliver implementation strategies to promote and sustain evidence-based management in acute care ED and paediatric inpatient areas.

Within the field of implementation science, de-implementation is an emerging field of research, only appearing in the literature within the last decade [38, 39]. There are very few studies that have examined sustainability of successful implementation interventions, with even less examining sustainability of de-implementing low-value care following completion of a successful behaviour change trial [20]. The majority of sustainability studies have been conducted in community and public health settings and not in acute care settings [29]. Few studies provide an a priori definition of sustainability [20] and often use established implementation frameworks to examine sustainability instead of using a sustainability framework [29]. Whilst evaluating sustainability frameworks is an agreed research priority, there is no consensus within the literature on which sustainability framework is the most suitable to inform sustainable evidence-based practice changes in acute care settings. This could be attributed to the dynamic nature of sustainability and the nature of acute care areas and is likely to evolve over the coming years [29–31].

This study is novel and innovative as it examines the sustainability of a successful de-implementation intervention in the acute care setting, two- years following removal of the study supports. We will report sustainability of clinician behaviour changes and sustainment of evidence-based interventions to de-implement low-value care. Whilst there is no consensus on when implementation phases end and when the sustainability period commences [19, 20, 30], two- years was deemed an appropriate timeframe to assess sustainability in an ED and acute inpatient setting. We have an ‘a priori*’* definition of sustainability and are using an established sustainability framework which has been deemed as relevant and useful when exploring sustainability in acute care settings [23, 29]. As such, our study will contribute to our understanding of sustainability of de-implementing low-value practices in paediatric acute care settings, and advancing the science of sustainability research.

## Data Availability

Not applicable.

## Abbreviations

ACU: Australian Catholic University
CXR: Chest X-ray
ED: Emergency Department
GLM: Generalised Linear Mixed
HREC: Human Research Ethics Committee
ICU: Intensive Care Unit
KT: Knowledge Translation
MCRI: Murdoch Children’s Research Institute
NHMRC: National Health and Medical Research Council
PI: Principal Investigator
PREDICT: Paediatric Research in Emergency Departments International Collaborative Knowledge Translation
REDCap: Research Electronic Data Capture
